# Comparative Transcriptome Profiling Reveals Different Expression Patterns in *Xanthomonas oryzae* pv. *oryzae* Strains with Putative Virulence-Relevant Genes

**DOI:** 10.1371/journal.pone.0064267

**Published:** 2013-05-29

**Authors:** Fan Zhang, Zhenglin Du, Liyu Huang, Casiana Vera Cruz, Yongli Zhou, Zhikang Li

**Affiliations:** 1 Institute of Crop Sciences/National Key Facility for Crop Gene Resources and Genetic Improvement, Chinese Academy of Agricultural Sciences, Beijing, China; 2 International Rice Research Institute, Metro Manila, The Philippines; 3 Beijing Institute of Genomics, Chinese Academy of Sciences, Beijing, China; Virginia Tech, United States of America

## Abstract

*Xanthomonas oryzae* pv. *oryzae* (*Xoo*) is the causal agent of rice bacterial blight, which is a major rice disease in tropical Asian countries. An attempt has been made to investigate gene expression patterns of three *Xoo* strains on the minimal medium XOM2, PXO99 (P6) and PXO86 (P2) from the Philippines, and GD1358 (C5) from China, which exhibited different virulence in 30 rice varieties, with putative virulence factors using deep sequencing. In total, 4,781 transcripts were identified in this study, and 1,151 and 3,076 genes were differentially expressed when P6 was compared with P2 and with C5, respectively. Our results indicated that *Xoo* strains from different regions exhibited distinctly different expression patterns of putative virulence-relevant genes. Interestingly, 40 and 44 genes involved in chemotaxis and motility exhibited higher transcript alterations in C5 compared with P6 and P2, respectively. Most other genes associated with virulence, including exopolysaccharide (EPS) synthesis, *Hrp* genes and type III effectors, including *Xanthomonas* outer protein (Xop) effectors and transcription activator-like (TAL) effectors, were down-regulated in C5 compared with P6 and P2. The data were confirmed by real-time quantitative RT-PCR, tests of bacterial motility, and enzyme activity analysis of EPS and xylanase. These results highlight the complexity of *Xoo* and offer new avenues for improving our understanding of *Xoo*-rice interactions and the evolution of *Xoo* virulence.

## Introduction

The gram-negative plant pathogenic *Xanthomonas oryzae* pv. *oryzae* (*Xoo*) is the causal agent of bacterial blight disease on rice [1]. Bacterial blight is the most serious bacterial disease of rice in tropical Asian countries where high-yielding rice cultivars are often highly susceptible, and it has the potential to reduce rice yields by as much as 50% [2]. The complete genome sequences have been published for Japanese race 1 [3], Korean race 1 [4], and PXO99^A^, a 5-azacytidine-resistant derivative of the Philippines’ race 6 [5]. These genomes have helped to elucidate the molecular interactions between a pathogen and a monocotyledonous plant and have greatly advanced the understanding of the molecular interactions between rice and *Xoo*. Several virulence-related factors have been identified, such as the hypersensitive response and pathogenicity (*hrp*) genes [6], type III (T3) effectors [7], [8], genes associated with the production of exopolysaccharides (EPS), and genes associated with motility and extracellular enzymes [9], [10].

The type II (T2S) and type III (T3S) secretion systems are important for the virulence of *Xoo*. The T3S system, encoded by *hrp* genes, plays an important role in interactions between *Xoo* and rice by injecting T3 effectors into plant cells, whereas the T2S system may play a role in the secretion of other virulence factors, such as extracellular enzymes like xylanase [11]. The T3S system is transcriptionally induced in certain minimal media and in plants [12], and the ompR-type response regulator HrpG, which is activated by unknown plant signals, controls the genome-wide regulon, including hrps, T3 effectors and putative virulence genes [13].

The collection of T3 effectors in *Xanthomonas* are designed as *Xanthomonas* outer proteins (Xop). Sixteen and 18 candidate Xop effectors were identified in *Xoo* strains MAFF311018 and PXO99^A^, respectively. Among them, XopZ_PXO99_ was demonstrated to contribute to the virulence of *Xoo* strains [14], [15]. Besides *Xop* genes, there is another important type T3 effector, the transcriptional activator-like (TAL) effectors in *Xoo*, which contain a central repeat domain in which amino acids 12 and 13 [known as the repeat variable diresidue (RVD)] of each repeat, and have been shown to transcriptionally activate the corresponding host genes for host disease susceptibility or resistance by recognizing and binding specific DNA sequences within the promoters of host target genes with RVDs [16]–[20].

The transcriptional regulation of putative virulence-relevant genes is critical to *Xoo* for infection and proliferation in rice varieties. Although the complete genome sequences of three *Xoo* strains from Asia and a draft genome sequence from Africa have been analyzed [3]–[5], [21], so far only microarray analysis has revealed that a greater number of *Xoo* genes are differentially expressed in XOM2 relative to PSB [22], and little is known about the transcriptome patterns of different strains. Analysis and comparisons of gene expression profiles in different strains will provide new insight into the pathogen’s virulence strategies. Here, we report the transcriptional expression profiling of genes involved in the virulence of three *Xoo* strains, one from China and two from the Philippines, induced on XOM2 minimal media.

## Materials and Methods

### Bacterial Culture and Isolation of Total RNA

*Xoo* strains PXO99 (Philippine race 6, P6), PXO86 (Philippine race 2, P2), and GD1358 (China race 5, C5) were used for this experiment. Cells were grown at 28°C with shaking at 200 rpm in nutrient-rich PSB (10 g/liter of peptone, 10 g/liter of sucrose, 1 g/liter of L-glutamic acid, monosodium salt) until OD_600_ equaled 2.0, washed twice, and immediately transferred into XOM2 for 16 h. XOM2 consists of 0.18% xylose sugar, 670 µM D, L-methiomine, 10 mM sodium L(+)-glutamate, 14.7 mM KH_2_PO_4_, 40 µM MnSO_4_, 240 µM Fe (III) EDTA and 5 mM MgCl_2_, pH 6.5 (Tsuge et al., 2002). Bacterial cells were washed twice prior to being harvested and snap-frozen in liquid nitrogen. Total RNA was extracted from each *Xoo* sample with RiboPure™-Bacteria kit and quantified by Qubit RNA assay kit (both from Applied Biosystems). The RNA integrity was checked with Agilent 2100 Bioanalyzer (Agilent Technologies).

### Pathogenicity Assays and Growth Curve of *Xoo*

**Figure 1:**
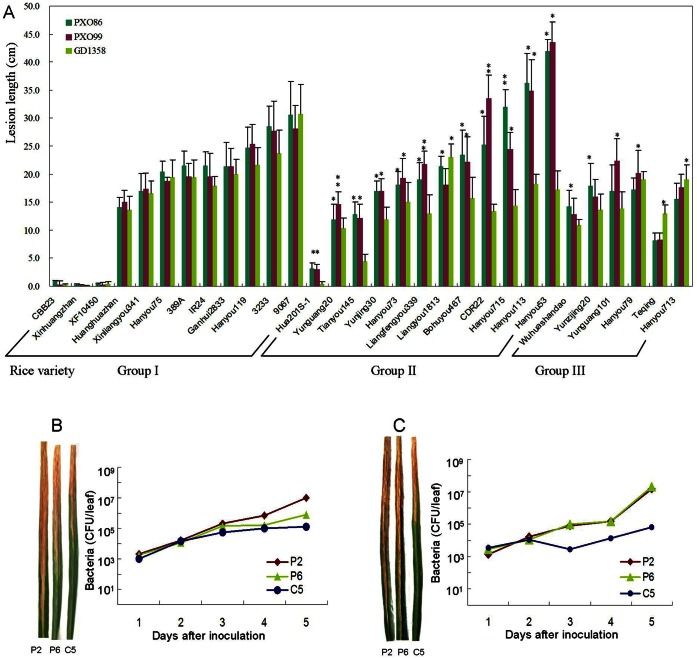
Lesion length of rice varieties inoculated by *Xanthomonas oryzae* pv. *oryzae* (*Xoo*) strains and growth curve of *Xoo*. (A) Lesion length of 30 rice varieties inoculated by three *Xoo* strains. * and ** represent significant difference at P<0.05 and 0.001, respectively. (B) Left: Hanyou715 exhibited different lesion lengths after infection with *Xoo* strains PXO99 (P6) and PXO86 (P2) from the Philippines and GD1358 (C5) from China. Right: growth of P2, P6 and C5 in Hanyou715. (C) Left: Hanyou53 exhibited different lesion lengths after infection with P2, P6 and C5. Right: growth of P2, P6 and C5 in Hanyou715. CFUs indicates the colony-forming units.

Thirty rice varieties (Figure 1A) were used to evaluate the pathogenicity of *Xoo* strains P6, P2 and C5. The plants were grown in the screenhouse of the Institute of Crop Sciences, Chinese Academy of Agricultural Sciences, Beijing, China in the summer of 2011. For evaluating bacterial blight resistance, seeds of the rice varieties were sown in the seedling nursery and 30-day-old seedlings were transplanted in the screenhouse with 9 plants in each row at spacing of 20×17 cm. At the tillering stage (plant age was 65 days), four to five of the uppermost leaves of each plant were inoculated with *Xoo* strains by the leaf-clipping method [23]. Inoculum of each race was prepared by suspending the bacterial mass in sterile water at a concentration of 10^8^ cells ml^−1^. Five central plants of each line were inoculated with each race for three replications. The lesion lengths (LL) were measured on all inoculated leaves 2 weeks after inoculation when lesions became obvious and stable in the susceptible variety IR24, and the average LL of each plant was calculated according to its three longest lesions. Evaluation of the resistance level of each variety was based on the average LL of 15 plants. LL≦1 cm, 1 cm ≦LL<5 cm, 5 cm≦LL<10 cm, 10 cm≦LL<15 cm, 15 cm≦LL<20 cm, and LL≥20 cm represent highly resistant, resistant, moderately resistant, moderately susceptible, susceptible and highly susceptible, respectively.

The growth curve of *Xoo* was produced according to the method described by Song et al. [24]. Five inoculated leaves of Hanyou715 and Hanyou53 from three plants were collected at 1, 2, 3, 4 and 5 days post-inoculation (dpi). The collected leaves were immediately frozen in liquid nitrogen, and then kept at −70°C. For each time point, the bacterial populations were determined by grinding three leaves separately, plating the resulting extract on potato sucrose agar media containing 200 µM azacytidine, and counting colony-forming units (CFUs) after 48 h at 28°C.

### Library Preparation and Illumina Sequencing

The ribosomal RNA (rRNA) was removed from 3 µg of total RNA with Ribo-Zero™ Magnetic kit for Gram-Negative Bacteria (EpicentreBio) by the manufacturer’s instructions. The RNA library was constructed according to the TruSeq™ RNA Sample Preparation kit (Illumina) with minor modification. Briefly, RNA was fragmented and the first cDNA strand was synthesized by using the random hexamers and SuperScript II Reverse Transcriptase (Invitrogen), then RNA template was removed and a replacement strand was synthesized to generate double-stranded (ds) cDNA. After end repair and 3′ end adenylation, the indexed adapter was ligated with the dsDNA. Fragments of 300∼350 bp were excised and enriched by PCR for 12 cycles. The yield and size distribution of PCR products were checked by QUBIT and Agilent 2100 Bioanalyzer respectively. The produced libraries were performed cluster generation on cBot and sequenced on HiSeq 2000 platform (illumina) with 100 bp paired-end reads by CapitalBio Corporation, Beijing, China. Illumina Casava(version 1.7) was used for basecalling, then all the sequencing data was processed by removing sequencing adapters for further analysis.

### Analysis of Illumina Sequencing

The analysis of RNA-seq sequencing data was performed as described by Zhao et al. [25] (Figure S1). All the tags mapped to reference sequences by Burrows-Wheeler Aligner [26], [27] with a maximum of five nucleotide mismatch. For gene expression analysis, the value of reads per kilo bases per million reads (RPKM) [28] was calculated. DEGseq [29] was applied to identify differentially regulated genes between two samples using the two classes unpaired MA-plot-based method to detect and visualize gene expression difference with significant *P* values less than 0.001. The whole genome sequence of *Xoo* was downloaded from the National Center for Biotechnology Information (www.ncbi.nlm.nih.gov), and coding regions were annotated according to the annotated protein data sets of *Xoo* strain PXO99^A^.

### Validation of Expression Patterns of DEGs Using Quantitative Real-time RT-PCR

To validate the results of the Illumina sequencing experiment, a subset of differentially expressed genes (DEGs) were verified by quantitative real-time RT-PCR (qRT-PCR). An independent set of cell cultures of the three *Xoo s*trains were cultured following the same protocol as for the Illumina analysis. QRT-PCR followed the methods described by Swarbrick et al. [30]. The sequence of each gene was obtained from the *X. oryzae* pv. *oryzae* PXO99^A^ database (http://www.ncbi.nlm.nih.gov), and the sequences from each gene were used for designing primers by Primer 5 software (http://frodo.wi.mit.edu/) (Table S1). RNA samples from three independent replicates for each treatment were pooled before cDNA synthesis. Thirty-three *Xoo* genes were tested in 50 µl reactions using the SYBR® Green PCR Master Mix kit (Applied Biosystems, CA, USA) following the manufacturer’s protocol. The correlation coefficient between the qRT-PCR and RNA-Seq results was calculated.

### Motility Analysis

Fresh colonies from PS agar plates were stabbed into swarm plates composed of 0.03% (wt/vol) Bactopeptone, 0.03% yeast extract, and 0.3% agar. The inoculated cells were cultured at 28°C and examined for bacteria swarming away from the inoculation site at 12, 24, 48 and 72 h after inoculation [31]. This study was repeated three times for reproducibility.

### Quantitative Determination of EPS and Xylanase Activity

The fresh colonies of *Xoo* strains were grown at 28°C with shaking at 200 rpm in nutrient-rich PSB until the OD_600_ equaled 2.0. They were then washed twice and immediately transferred into XOM2. After growth for 16 h, the bacterial cultures were collected and supernatants were prepared by centrifugation at 5000 rpm for 10 min. The extracellular xylanase was measured by using 4-O-methyl-D-glucurono-D-xylan-Ramezol Brilliant Blue R (RBB-xylan; Sigma Co.) according to the methods described by Biely et al. [32]. The production of EPS was determined according to the methods described by He et al. [33].

## Results

### Pathogenicity Testing of *Xoo* Strains

To evaluate the pathogenicity of P6, P2 and C5, 30 rice varieties, including 29 recently developed varieties in China, and a cultivar susceptible to all Philippine *Xoo* races from the International Rice Research Institute (IRRI), IR24, were inoculated at the tillering stage. Also included were CBB23, Xinhuangzhan, and Hua201S-1, which carry the bacterial blight resistance gene *Xa23*, a single completely dominant resistance gene identified from wild rice species of *Oryza rufipogon* [34], [35]; *Xa21* from the wild rice strain XF10450 [36] and *O*. *longistaminata* [37], and *Xa7* from IRBB7 [38]. Only four varieties were resistant to the three *Xoo* strains. Among them, three varieties including CBB23, Xinhuanzhan and XF10450, exhibited a typical hypersensitive reaction (HR) with less than 0.5 cm LL. The LL of Hua201S-1 inoculated with P6, P2 and C5 was 3.0±1.1 cm, 3.1±0.9 cm and 0.4±0.4 cm, respectively. Except for Teqing, which was moderately resistant to P6 and P2 and susceptible to C5, the other 25 varieties displayed a moderate or high susceptibility to the three strains with LLs ranging from 12.1 to 43.5 cm.

The 30 rice varieties were placed in three groups depending on the LL phenotypes exhibited after inoculation (Figure 1 A). Group I, which showed LL ranging from 0.2±0.1 to 28.1±5.9 cm, 0.3±0.1 to 30.6±4.1 cm and 0.1±0.1 to 30.7±5.3 cm against P6, P2 and C5, respectively, consisted of 12 varieties: CBB23, Xinhuanzhan, XF10450, Huanghuazhan, Xinliangyou341, Hanyou75, 389A, IR24, Ganhui2833, Hanyou119, 3233, and 9067. There were no significant differences among LLs within the same variety when inoculated with the three *Xoo* strains. Group II, with LL ranging from 3.0±1.1 to 43.5±2.1 cm, 3.1±0.9 to 42.0±3.6 cm and 0.4±0.4 to 32.1±2.4 cm against P6, P2 and C5, respectively, also consisted of 12 varieties: Hua2018, Yunguang20, Tianyou20, Tianyou145, Yunjing30, Hanyou73, Liangfengyou339, Bohuyou813, CDR22, Hanyou715, Hanyou113, and Hanyou53. The LLs of the same variety were significantly longer when inoculated with two of the *Xoo* strains than when infected by the third strain. Eleven of the varieties had significantly longer LLs when infected by P6 or P2 than when infected by C5. The exception was the variety Liangyou1813 (Figure 1A). Bacterial growth curve analysis indicated that the growth of P2 and P6 increased by more than three-fold compared with C5 in Hangyou715 36 hr post-inoculation, and the growth of P2 and P6 increased by more than 20-fold compared with C5 in Hangyou53 36 h post-inoculation (Figure 1B, C).

Group III had six varieties. The LLs caused by one strain were significantly longer than when infected by the other two strains. The LLs of Wuhuashandao and Yunzijing20 when infected by P6 were significantly longer than when infected by P2 and C5, and the LLs of Yunguang101 and Hanyou79 when infected by P2 were significantly longer than when infected by P6 and C5. However, the LLs of Teqing and Hanyou713 when infected by C5 were significantly longer than when infected by P6 and P2.

Our results indicated that the virulence of P2 was similar with that of P6 when infecting 21 rice varieties, and the LLs of five and four varieties were significantly longer and shorter against P6 than against P2, respectively. However, there was no significant difference between the virulence of C5 and P6 when infecting 14 rice varieties, C5 was significantly weaker than P6 when infecting 13 rice varieties, and C5 was stronger than P6 when infecting two varieties.

### Mapping of mRNA-seq Reads and Statistical Testing to Detect Differentially Expressed Genes

**Figure 2:**
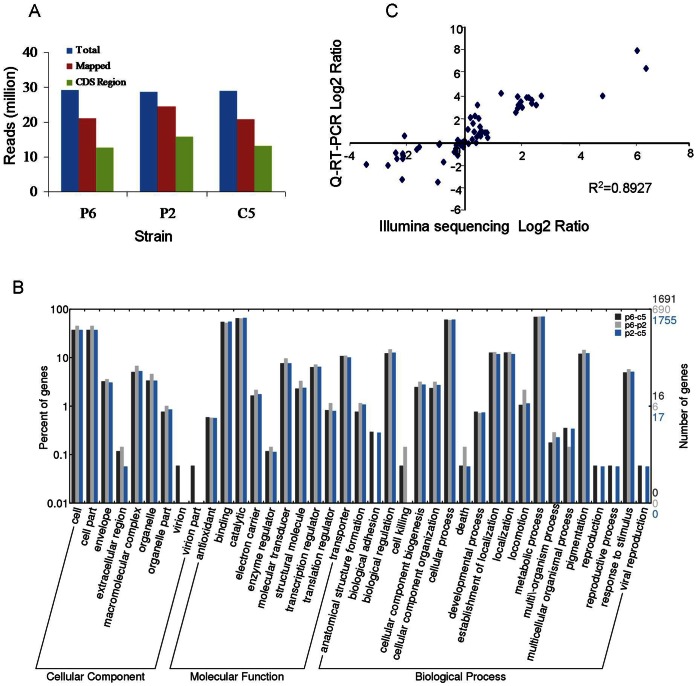
Overview of mRNA-seq data and mapping to the *Xanthomonas oryzae* pv. *oryzae* (*Xoo*) PXO99A genome. (A) Total number of mRNA-seq reads mapped in each *Xoo* strain library. (B) Histogram presentation of gene ontology classifications. The results are summarized in three categories: cellular components, molecular functions and biology processes. The right y-axis indicates the number of genes in a category. The left y-axis indicates the percentage of a specific category of genes in that main category. (C) Comparison of transcription measurements by Illumina sequencing and qRT-PCR assays. The correlation coefficient (R2) between the two datasets is 0.8927.

To compare the transcriptome profile of *Xoo* strains with different virulence levels, RNA sequencing libraries were constructed for P6, P2 and C5. Each library generated about 28.6 to 29.2 million reads, which were mapped to the PXO99^A^ genome sequence (NCBI Reference Sequence: NC_010717.1) with 78.9%, 80.1% and 81.6% matched reads to NCBI annotated gene regions, respectively (GEO database: accession number GSE44215). There were 5,083 protein-coding genes predicted in the genome of PXO99^A^ by genome analysis [39]. In total, 4,781 transcripts were identified in this study. With a threshold of more than five reads mapped to the CDS regions of a given gene in each sample, a total of 4,605, 4,503 and 4,467 protein-coding genes were detected in P6, P2 and C5, respectively (Figure 2A).

The R-package DEGseq [29] was used to identify DEGs. The list of genes with significantly different expression levels between two strains was refined using the criterion of *P* value <0.001 in *t* tests, resulting in 1,151, 3,076 and 3,112 DEGs when P6 was compared with P2, P6 was compared with C5, and P2 was compared with C5, respectively (Table S2, S3, S4). And all the DEGs between each of the comparisons shared 782 genes in common (Figure S2).

Gene ontology (GO) assignments were used to classify the function of the DEGs. Based on sequence homology, the DEGs can be categorized into 39 functional groups (Figure 2B). In the three main categories, cellular component, molecular function and biological process, of GO classifications, “cell part”, “catalytic” and “metabolic process” terms are dominant, respectively. We also noticed a high-percentage of genes from categories of “cell”, “binding” and “cellular process” as well as a few genes from “reproduction”, “reproductive process” and “viral reproduction”.

### Validation of Expression Patterns by qRT-PCR

To validate the Illumina sequencing results, qRT-PCR was used to independently assess expression levels for 33 genes involved in motility, the T3S system and T3 effectors (genes and primer sets used are shown in Table S1; the results of qRT-PCR are shown in Figure S3). RNA samples that were used in the Illumina sequencing experiment as well as RNA samples extracted from three additional replicate sets of cultures were used as templates. There was a good correlation between the qRT-PCR and the mRNA-seq results (correlation coefficient was 0.8297) (Figure 2C). Although the amplitude of gene expression fold change between the two techniques is different, as might be expected since qRT-PCR is not a reliable measure of quantitative differences, the general trend of gene expression is consistent.

### Differential Expression of Two-component Systems between *Xoo* Strains

**Figure 3:**
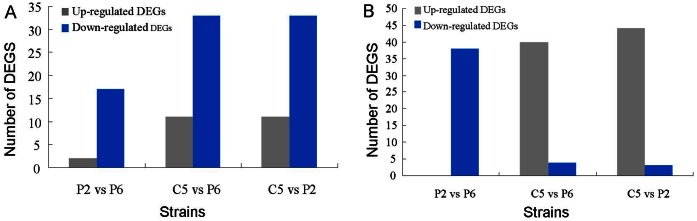
Differentially expressed genes (DEGs) between three *Xanthomonas oryzae* pv. *oryzae* strains, PXO99 (P6) and PXO86 (P2) from the Philippines and GD1358 (C5) from China. (A) Genes related to the two-component systems. (B) Genes related to chemotaxis and motility.

Two-component systems (TCSs) are widespread signal transducers in prokaryotes that serve as a basic stimulus-response coupling mechanism to allow organisms to sense and respond to changes in many different environmental conditions [40]. They typically consist of a membrane-bound histidine kinase that senses a specific environmental stimulus and a corresponding response regulator that mediates the cellular response [41]. In this study, 55 transcripts associated with TCSs were identified in three *Xoo* strains (Table S5), and DGE analysis revealed that the transcriptome profile of C5 was considerably different from P6 and P2, which exhibited similar expression patterns. Nineteen TCSs differentially expressed in P2 compared with P6. Among them there were two and 17 significantly up- and down-regulated genes, respectively. Forty-four genes differentially expressed in C5 compared with P6; 11 and 33 were significantly up- and down-regulated genes in C5, respectively. Similarly, of the 44 TCSs differentially expressed in C5 compared with P2, 11 and 33 were significantly up- and down-regulated genes in C5, respectively (Figure 3A).

The TCS transcriptome profiling of three *Xoo* strains revealed two interesting aspects. First, the expression pattern of the RpfC/RpfG two-component regulatory system associated with quorum sensing (QS) was different between P2, P6 and C5 (Table 1). Genetic and genomics evidence suggest that *Xoo* might use the diffusible signal factor (DSF) QS system to regulate virulence factor production [33]. QS is a complicated bacterial group behavior for producing, sensing and responding to multifarious chemical signals, which increases their chances of survival and propagation, and it provides bacterial pathogens an obvious competitive advantage over their hosts in pathogen-host interactions [42]. The RpfC/RpfG two-component regulatory system is implicated in sensing and responding to DSF perception and signal transduction [43], [44]. *RpfC* negatively controls DSF biosynthesis by binding to *rpfF* at a low cell density [33]. Several GGDEF, EAL and HD-GYP domain proteins of *X. campestris* pv. *campestris* (*Xcc*) are hypothesized to compose a network of signal transduction systems for response to different environmental cues to modulate the level of the second messenger cyclic di-GMP [45]. Our results indicated that a TCS regulatory protein with a HD-GYP domain (PXO_00476) had significantly down-regulated expression in C5 compared with P6 and P2. However, a TCS regulatory protein with a GGDEF domain (PXO_00466) had significantly up-regulated expression in C5 compared with P6 and P2. Moreover, cyclic di-GMP phosphodiesterase A (PXO_00058) had significantly up-regulated expression in C5 compared with P6 and P2.

**Table 1:** Expression profiles of *rpf* and *rax* genes in three *Xanthomonas oryzae* pv. *oryzae* strains.

Locus ID	P6 vs. P2*			P6 vs. C5			P2 vs. C5			Gene Name/Function
	Log2 fold-change^a^	P value	Signature^b^	Log2 fold-change	P value	Signature	Log2 fold-change	P value	Signature	
PXO_00069	0.0842	1.49E-06	True	0.1886	9.48E-24	True	0.1044	5.94E-09	True	*RpfC*
PXO_00070	0.1302	6.30E-12	True	−0.1081	1.67E-08	True	−0.2382	4.04E-38	True	*RpfG*
PXO_00073	0.1078	0.0008	True	0.3191	1.10E-19	True	0.2113	3.77E-10	True	*RpfD*
PXO_00084	−0.1047	0.0551	False	−0.1772	0.0018	False	−0.0725	0.1674	False	*RpfE*
PXO_00068	0.0615	0.0128	False	0.3166	2.69E-31	True	0.2551	7.03E-23	True	*RpfF*
PXO_00067	−0.0227	0.291	False	0.1906	5.82E-16	True	0.2133	4.79E-22	True	*RpfB*
PXO_00064	−0.4466	3.77E-126	True	−0.5352	1.44E-167	True	−0.0886	1.61E-07	True	*RpfA*
PXO_00058	0.3422	9.37E-73	True	−1.2474	0	True	−1.5897	0	True	C-di-GMP phosphodiesterase A
PXO_00476	0.0542	0.163	False	0.4295	8.98E-23	True	0.3753	1.81E-19	True	Two-component system regulatory protein with HD-GYP domain
PXO_00466	0.724	2.01E-230	True	−1.7169	0	True	−2.4409	0	True	Two-component system regulatory protein with GGDEF domain
PXO_02944	−0.0315	0.1155	False	0.0446	0.0366	False	0.0761	0.0001	True	Two-component system regulatory protein with GGDEF and EAL domains
PXO_01585	−0.0217	0.518	False	1.4111	4.50E-212	True	1.4328	8.08E-248	True	PhoPQ-regulated protein
PXO_04467	−0.097	0.2354	False	0.403	1.78E-05	True	0.5	9.12E-09	True	*RaxH*
PXO_02837	−0.014	0.7173	False	0.4235	7.21E-22	True	0.4376	4.37E-26	True	*RaxH2*
PXO_02836	−0.0124	0.4796	False	0.2808	3.10E-47	True	0.2932	7.58E-58	True	*RaxR2*
PXO_04469	0.2741	0.0004	True	0.4216	4.36E-07	True	0.1475	0.0736	False	*RaxR*
PXO_04478	−0.0551	0.2428	False	1.3968	7.03E-106	True	1.4519	7.07E-131	True	*RaxA*
PXO_04477	−0.0533	0.1293	False	1.2391	1.18E-157	True	1.2924	2.68E-196	True	*RaxB*
PXO_02621	−0.0355	0.0845	False	0.2162	1.48E-21	True	0.2518	1.96E-32	True	*RaxC*
PXO_02134	−0.0819	0.1335	False	0.5034	2.57E-15	True	0.5853	4.15E-23	True	*RaxP*
PXO_02135	−0.0918	0.0126	False	0.7017	4.51E-56	True	0.7936	1.72E-82	True	*RaxQ*
PXO_04479	−0.1778	2.23E-07	True	0.9684	8.61E-107	True	1.1462	7.08E-178	True	*RaxST*

a The fold change of gene expression is represented by a log2 ratio. A log2 ratio of 0.8 is equivalent to a 1.75 fold relative increase in expression.

b ‘Ture’ means significant differential expression (p-value <0.001), and ‘False’ represents the opposite.

* P2, P6 and C5 represent PXO86, PXO99 and GD1358, respectively.

Second, significant up-regulation of the phoPQ-regulated protein (PXO_01585) was detected in P6 and P2 when compared with C5 (Table 1). This protein is not only required for AvrXA21 activity, but also controls virulence through the regulation of *hrpG* gene expression [46]. It also regulates numerous cellular activities in *Salmonella* and other species as a master regulator of virulence [47], [48]. AvrXA21 requires a regulatory TCS called RaxRH to regulate expression of 10 *rax* (required for AvrXA21 activity) genes. Our results indicated that *raxH* (PXO_04467), *raxH2* (PXO_02837), *raxR* (PXO_04469) and *raxR2* (PXO_02836) were significantly up-regulated in P6 and P2 compared with C5. Additionally, *raxA* (PXO_04478), *raxB* (PXO_04477), *raxC* (PXO_02621), *raxP* (PXO_02134), *raxQ* (PXO_02135) and *raxST* (PXO_04479) were also significantly up-regulated (Table 1). However, there was no significant difference in the expression of the phoPQ-regulated protein and the *rax* genes, except for *raxR* and *raxST* between P6 and P2. This suggests the expression pattern of genes involved in AvrXA21 activity and *hrpG* expression exhibited by C5 was different from P6 and P2.

### A set of Genes Possibly Related to Chemotaxis and Bacterial Motility had Significantly Up-regulated Expression Levels in C5

**Figure 4:**
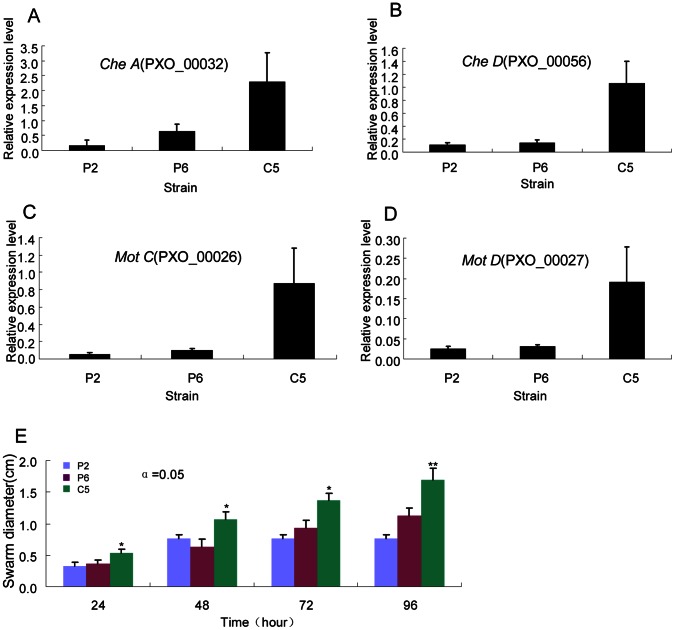
The expression patterns of genes associated with motility and swarming motility of *Xanthomonas oryzae* pv. *oryzae* (*Xoo*) strains PXO99 (P6) and PXO86 (P2) from the Philippines and GD1358 (C5) from China. (A) The expression patterns of cheA. (B) The expression patterns of cheD. (C) The expression patterns of motC. (D) The expression patterns of motD. The gene expression level (arbitrary units) was normalized using 16sRNA as an internal reference. The gene expression level was quantified by real-time RT-PCR. (E) Swarming motility of *Xoo* on plates. * and ** represent significant difference at P<0.05 and 0.001, respectively.

The number of DEGs involved in chemotaxis and motility were differentially expressed between the three strains and exhibited interesting expression patterns (Table S6; Figure 3B). These genes mainly included chemoreceptors, chemotaxis proteins, twitching motility proteins, flagellar motor proteins, pilus biogenesis proteins and pilus assembly proteins. Of the differentially expressed genes, 38 genes had down-regulated expression levels in P2 when compared with P6. In addition, 40 genes had up-regulated expression levels and four genes had down-regulated expression levels in C5 when compared with P6. Finally, 44 genes had up-regulated expression levels and three genes had down-regulated expression levels in C5 when compared with P2. In general, the structural genes encoding motility systems are clustered within large transcriptional units allowing co-regulation of their expression [49]. We found that the expression of the chemoreceptor glutamine deamidase *CheA* (PXO_00032) increased greater than 2.89-fold in C5 when compared with P6 and P2 (Figure 4A). Consistent with this finding, 15 and 17 genes involved in the encoding and synthetic metabolism of chemotaxis proteins, including *CheD* (PXO_00056) (Figure 4B), were up-regulated in C5 compared with P6 and P2, respectively. Additionally, many *pil* genes involved in bacterial movement [50], including *pilG* (PXO_01602), *pilH* (PXO_01603), *pilL* (PXO_01607), *pilV* (PXO_01321), *pilX* (PXO_01323), *pilY1* (PXO_01324) and *pilZ* (PXO_00049), were also up-regulated in C5 compared with P6 and P2. In addition, four genes encoding flagellar motor proteins, including *MotA* (PXO_03068), *MotB* (PXO_03067), *MotC* (PXO_00026) and *MotD* (PXO_00027) (Figure 4C, D), and two genes encoding twitching motility proteins, including PXO_01994 and PXO_01993, were significantly up-regulated in C5.

Swarm plate analysis displayed that the swimming diameter of C5 was significantly larger than those of P6 and P2, and they tended to form larger swarming colonies at 24, 48, 72 and 96 h post-inoculation (Figure 4E). This confirms that the motility of C5 was significantly stronger as reflected by the enhanced expression levels of many genes encoding chemotaxis proteins, pil proteins and flagellar motor proteins.

### Differential Expression of a Gum Gene Cluster Involved in EPS Synthesis

The *gum* gene cluster involved in EPS synthesis functions as a virulence determinant in *Xanthomonas* [51]. EPS synthesis in *Xcc* is directed by genes within the *gum* cluster, which contains 12 genes and has a major promoter upstream of the first gene, *gumB* [52]. Similarly, the *Xoo gum* cluster is composed of 14 ORFs that constitute an operon expressed from a promoter located upstream of *gumB*, but the cluster also has internal promoters upstream of *gumG*, *gumH* and *gumM* [46], [53]. In our study, 12 gum genes were differentially expressed in C5 compared with P6 and P2. Except for the *gumB* (PXO_01391) up-regulated gene, the other 11 genes from *gumC* to *gumM* (PXO_01392-PXO_01403) were all down-regulated (Table S7). By contrast, five genes (*gumD*, *gumH*, *gum K*, *gumL*, and *gumM*) were up-regulated in P2 compared with P6. In addition, two genes encoding xylanase (PXO_03864 and PXO_04558) were down-regulated in C5 compared with P6 and P2, and PXO_04558 also had down-regulated expression in P2 compared with P6.

**Figure 5:**
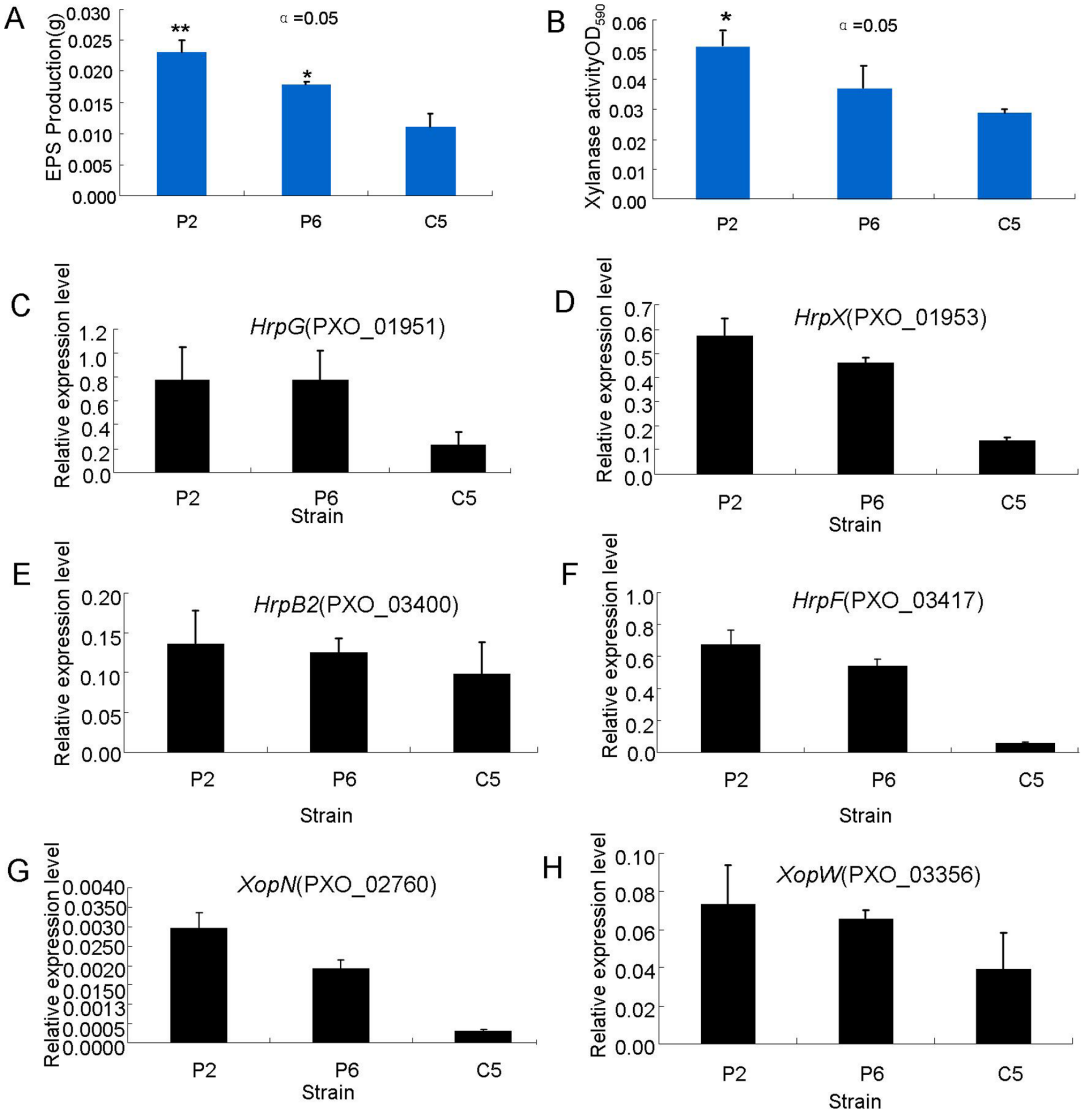
The activity of exopolysaccharides (EPS) and xylanase, and the expression patterns of *hrp* genes and *Xop* genes of *Xanthomonas oryzae pv. oryzae* strains PXO99 (P6) and PXO86 (P2) from the Philippines and GD1358 (C5) from China. (A) EPS activity. (B) Xylanase activity. * and ** represent significance difference at P<0.05 and 0.001, respectively. (C) The expression patterns of *hrpG*. (D) The expression patterns of *hrpX*. (E) The expression patterns of *hrpB2*. (F) The expression patterns of *hrpF*. (G) The expression patterns of *xopN*. (H) The expression patterns of *xopW*. The gene expression level (arbitrary units) was normalized using 16sRNA as an internal reference. The gene expression level was quantified by real-time RT-PCR.

Interestingly, *xanA* (PXO_03174), *xanB* (PXO_03173), *wxoA* (PXO_03160), *wxoB* (PXO_03161), *wxoC* (PXO_05411), and *wxoE* (PXO_03158) were also down-regulated in C5 compared with P6 and P2. However, *xanA* (PXO_03174) and *xanB* (PXO_03173) were up-regulated in P2 compared with P6 and there was no difference in the expression levels of the other genes. In addition, two genes encoding xylanase (PXO_03864 and PXO_04558) were significantly down-regulated in C5 compared with P6 and P2. Activity analysis showed that EPS activity in P2 and P6 was significantly higher than that in C5 (Figure 5A), and the xylanase activity in P2 was significantly higher than that in P6 and C5 (Figure 5B).

Jeong et al. (2008) [54] reported that *RpfB*, *rpfC*, *rpfF*, and *rpfG* are important for the virulence of *Xoo* KACC10859, and that some virulence genes revealed the significantly reduced expression of genes related to exopolysaccharide (EPS) production (*gumG* and *gumM*), lipopolysaccharide (LPS: *xanA*, *xanB*, *wxoD*, and *wxoC*), xylanase (*xylB*), and motility (*pilA*) in the mutants *rpfB*, *rpfC*, *rpfF* and *rpfG* [54]. However, our results indicated that the activities of EPS and xylanase were reduced in C5, but that the bacterial motility increased when *rpfB* (PXO_00067), *rpfC* (PXO_00069) and *rpfF* (PXO_00068) were down-regulated and *rpfG* (PXO_00070) was up-regulated.

### *Hrp* Genes and T3 Effectors had Significantly Up-regulated Expression Levels in P6 and P2

Knowledge of *hrp* genes in Xanthomonas arises mainly from studies of the *X. campestris* species [55]. *Hrp* genes are essential for pathogenicity in both *Xoo* and *Xoc*. *HrpF* has been found to be a putative type III translocon protein required for pathogenicity [56]. *HrpG* activates the expression of *hrpA* and *hrpX*. *HrpX* encodes a protein belonging to the AraC family of positive transcriptional activators and controls the expression of operons *hrpB* to *hrpF* as well as *avrXv3* and a number of putative virulence factors. We examined expression of *hrp* genes and tested the expression of several genes by qRT-PCR (Figure 5C, D, E, F; Figure S3). Interestingly, the expression of 29 genes associated with *hrp* genes (Table S8), including genes encoding *hrpF* (PXO_03417), *hrpG* (PXO_01951) (Figure 5C), the *hrpA* type III secretion outer membrane pore (PXO_03393) and *hrpX* (PXO_01953) (Figure 5D), were significantly down-regulated in C5 compared with P6 and P2. However, 14 of 29 genes were up-regulated in P2 compared with P6.

We also specifically examined the expression levels of T3 effectors. Fifteen *Xop* genes were significantly down-regulated in C5 when compared with P6 and P2, and 10 *Xop* genes were down-regulated in P6 when compared with P2 (Table 2). These effectors were expressed in a HrpX-dependent manner, suggesting the co-regulation of effectors and the T3S system. Consistent with the results of sequencing, the expression difference between *XopN* (PXO_02760) and *XopW* (PXO_03356) among the three strains was also confirmed by qPCR (Figure 5G, H). In addition, 11 and 12 genes encoding TAL effectors were down-regulated in C5 compared with P2 and P6, respectively. Among them, *pthxo1* (PXO_03922) increases bacterial populations in plants. However, six genes were down-regulated in P6 compared with P2, including *pthxo1* (Table 2).

**Table 2:** Expression profiles o*f xop* and *tal* genes in three *Xanthomonas oryzae* pv. *oryzae* strains.

Locus ID	P6 vs. P2*			P6 vs. C5			P2 vs. C5			Gene name
	Log2 fold-change^a^	P value	Signature^b^	Log2 fold-change	P value	Signature	Log2 fold-change	P value	Signature	
PXO_03413	−0.2477	2.76E-11	True	1.0869	2.75E-107	True	1.3346	7.51E-197	True	*XopF1*
PXO_03356	−0.0368	0.2331	False	1.7430	0	True	1.7798	0	True	*XopW*
PXO_03901	−0.0990	0.0003	True	−0.0550	0.0594	False	0.0440	0.1034	False	*XopQ*
PXO_03833	−0.2146	6.85E-34	True	1.9105	0	True	2.1251	0	True	*XopAD*
PXO_03819	−0.1194	6.94E-17	True	1.5300	0	True	1.6493	0	True	*XopR*
PXO_03702	−0.2793	2.69E-42	True	1.3885	0	True	1.6677	0	True	*XopX*
PXO_04172	−0.0505	0.1077	False	1.4076	5.39E-239	True	1.4582	1.57E-293	True	*XopV*
PXO_04866	−0.2214	1.63E-06	True	0.7567	2.32E-39	True	0.9781	3.90E-78	True	*XopY*
PXO_00236	−0.0307	0.1122	False	0.4611	3.44E-96	True	0.4918	5.42E-124	True	*XopU*
PXO_00234	0.0029	0.9379	False	1.7636	4.30E-243	True	1.7608	2.03E-272	True	*XopAA*
PXO_01625	−0.3258	1.74E-36	True	0.8774	5.20E-151	True	1.2032	0	True	*XopK*
PXO_01620	−0.0417	0.1091	False	2.0989	0	True	2.1406	0	True	*XopL*
PXO_02108	−0.3271	1.74E-32	True	1.8881	0	True	2.2152	0	True	*Xopc*
PXO_02107	−0.1448	1.44E-07	True	3.2766	0	True	3.4214	0	True	*XopP*
PXO_02170	0.5070	0.2176	False	3.7467	8.16E-07	True	3.2396	3.40E-05	True	*XopP*
PXO_02760	−0.2747	6.32E-98	True	1.9034	0	True	2.1780	0	True	*XopN*
PXO_00223	0.0561	0.3260	False	0.8741	2.15E-36	True	0.8180	2.88E-35	True	*Tal2a*
PXO_05718	−0.0344	0.5475	False	0.8400	5.01E-33	True	0.8744	2.13E-40	True	*AvrXa27/tal9c*
PXO_05714	−0.0528	0.1675	False	1.0656	3.08E-105	True	1.1183	1.78E-132	True	*Tal9b*
PXO_00572	−0.2600	9.03E-07	True	1.3748	6.02E-77	True	1.6347	1.09E-132	True	*Pthxo6/tal5b*
PXO_00546	−0.2838	0.0006	True	−0.1354	0.1287	False	0.1484	0.0633	False	*Tal6a*
PXO_02269	−0.0317	0.5266	False	1.8221	3.84E-138	True	1.8539	1.65E-162	True	*Tal9d*
PXO_06229	−0.8398	0.3118	False	−1.5387	0.0471	False	−0.6990	0.2682	False	*Tal8a*
PXO_00567	−0.2610	0.0002	True	0.8474	1.37E-21	True	1.1084	1.06E-43	True	*Tal5a*
PXO_01085	−0.6698	0.2400	False	−0.1058	0.8731	False	0.5640	0.3101	False	*Tal7b*
PXO_02272	−0.1400	0.0004	True	0.3954	8.41E-18	True	0.5354	1.04E-36	True	*Tal9e*
PXO_03922	−0.1242	0.0235	False	−0.0431	0.4625	False	0.0812	0.1330	False	*Pthxo7/tal1*
PXO_00318	−0.2166	0.0406	False	0.6454	6.10E-07	True	0.8620	2.12E-13	True	*Tal4*
PXO_00511	−0.4406	1.50E-25	True	0.0699	0.1499	False	0.5105	2.04E-33	True	*Tal3a*
PXO_00505	−0.2363	1.47E-13	True	1.5025	1.21E-238	True	1.7388	0	True	*Tal3b*
PXO_00227	−0.1228	0.0024	False	2.8134	0	True	2.9362	0	True	*Pthxo1/tal2b*
PXO_05609	0.0551	0.5097	False	1.8985	2.91E-55	True	1.8434	1.66E-57	True	*Tal6b*
PXO_02172	−0.1020	0.0532	False	−0.1076	0.0526	False	−0.0056	0.9127	False	*Tal9a*
PXO_06234	0.2147	0.7091	False	−0.6167	0.2401	False	−0.8314	0.1054	False	*Tal8b*
PXO_05633	−0.1844	0.7931	False	−1.1464	0.0675	False	−0.9620	0.0928	False	*Tal7a*

a The fold change of gene expression is represented by a log2 ratio. A log2 ratio of 0.8 is equivalent to a 1.75 fold relative increase in expression.

b ‘Ture’ means significant differential expression (p-value <0.001), and ‘False’ represents the opposite.

* P2, P6 and C5 represent PXO86, PXO99 and GD1358, respectively.

## Discussion

Bacterial blight occurs in most rice-growing areas of the world, and *Xoo* isolates from within and across Asia, Africa, and Australia show a great diversity of genotypes based on the polymorphisms of transposable elements, predominantly insertion sequences (IS), avirulence genes, rep/box elements, and other markers [57]. The great diversity of strains within *Xoo* undoubtedly reflects adaptation of the pathogen to the diversity of host genotypes as well as the diverse environmental conditions in which rice is grown. Our transcriptome profiling analysis revealed some interesting aspects of different *Xoo* strains from the Philippines and China with putative virulence-relevant genes *in* *vitro*.

First, a large set of genes associated with the expression of *Hrp* genes and T3 effectors were significantly up-regulated in P6 and P2. This finding suggests that *hrp* genes and genes encoding T3 effector expression may differ for these strains in rice varieties. In phytopathogenic bacteria, the T3S system is encoded by *hrp* genes for eliciting HR on non-host or resistant host plants and for pathogenesis on susceptible hosts [55]. More and more evidence demonstrates that Hrp proteins and TAL effectors play key roles in host immunity responses or facilitate nutritional or virulence processes in the pathogen. These may trigger a resistance response in plants that contains TAL effector recognition features (such as AvrXa27 and AvrXa10), some of which are critical for virulence (such as PthoXo1, PthoXo6 and PthoXo7), and others of which appear to have more moderate or contextual functions in virulence [58]. The genomic sequences of the published Philippine (PXO99^A^), Japanese (MAFF311018) and Korean (KACC10331) strains contain 19, 17 and 15 TAL effector genes, respectively, and the African strain BAI, may contain eight TAL effector genes [21]. However, the *X. oryzae* strains in the United States lack TAL effectors and exhibit weak pathogenicity and a severely limited range of host cultivars compared with the Asian and African *Xoo* strains [59].

Although Illumina data alone were not sufficient to decipher the complicated and repetitive nature of the TAL effector coding sequences in P2 and C5, our results indicate that the expression patterns of *hrp* genes and T3 effectors were significantly different between *Xoo* strains from China and the Philippines. Together with the phenotypes of rice varieties, growth curves in Hangyou715 and Hanyou53, and expression patterns of the pathogen, we speculated that the increased virulence of P6 and P2 compared with C5 when infecting some rice varieties was due to the differentially up-regulated expression of *hrp* genes and T3 effector genes, which might promote *Xoo* multiplication in rice plants. The finding that three TAL effectors targeting the OsSWEET family of sucrose transporters conferred an increased virulence to the weakly pathogenic USA *X. oryzae* strain supported our speculation [60]. So far, whether the divergence of T3 effectors of *Xoo* potentially correlate with the geographic origin and diversity of rice cultivars remains a mystery. Answering this question will be facilitated by the determination of the full genome sequence of more *Xoo* strains.

Second, a large set of genes encoding chemotaxis and proteins involved in bacterial motility were significantly up-regulated in C5. Motility over solid surfaces is an important bacterial mechanism that allows complex social behaviors and pathogenesis. In some plant-pathogen systems, flagella-driven chemotaxis plays a role in the early interactions with host plants, and motility enables foliar pathogens to reach internal sites in the leaves [61]. Moreover, the bacterial protein flagellin has been found to be a plant elicitor, and plants have a sensitive perception system for this protein [62], [63]. Even though the genes encoding Hrp proteins and T3 effectors were significantly down-regulated in C5, there was no significant difference in the LLs of 14 rice varieties infected by C5, P6 and P2. We hypothesize that this is due to C5’s stronger motility, which might *compensate for the weaker expression levels of* Hrp proteins and T3 effectors, and allows C5 to exhibit similar virulence levels with P2 and P6 in some rice varieties. Verdier et al. (2012) recently reported that the plant genetic background affected the level of virulence enhancement by *Xoo* TAL effectors [60]. This also provides a clue to why there was no significant difference in virulence levels among P2, P6 and C5 when they infected some rice varieties.

Our analysis of the *Xoo* transcriptomes based on deep transcriptome sequencing led to remarkable insights into the transcriptional landscape of this important model plant pathogen from different countries, and it offers new avenues for improving our understanding of the *Xoo*-rice pathogenic mechanism and the evolution of *Xoo* virulence. Further understanding of the roles of bacterial motility and TAL effectors in diverse plant genetic backgrounds would shed light or provide further insights into their roles in interaction with the host, especially when we include the differing or diverse genetic background of the rice varieties and the environment or ecosystem where the crop is grown.

## Supporting Information

Figure S1 The analysis workflow for RNA-seq data based on mapping reads to the reference genome sequence, quantifying the gene expressed, identifying DEGs and categorizing DEGs by Gene Ontology.
(PPT)

Figure S2 The Venn diagram of all the DEGs between each of the comparisons (P6 vs. C5, P6 vs. P2, P2 vs. C5). P6, P2 and C5 indicate *Xanthomonas oryzae* pv. *oryzae* strain PXO99, PXO86, and GD1358, respectively.
(PPT)

Figure S3 The expression patterns of 33 genes in three *Xanthomonas oryzae* pv. *oryzae* strains, PXO99 (P6) and PXO86 (P2) from the Philippines and GD1358 (C5) from China.
(PPT)

Table S1 Primer sequences for real-time PCR analysis of differentially regulated genes in *Xanthomonas oryzae* pv. *Oryzae.*
(XLS)

Table S2 Differentially expressed genes in *Xanthomonas oryzae* pv. *oryzae* strains from the Philippines, PXO99 (P6) and PXO86 (P2), when PXO86 is compared with PXO99.
(XLS)

Table S3 Differentially expressed genes in *Xanthomonas oryzae* pv. *oryzae* strains when strain GD1358 (C5) from China is compared with strain (P6) from the Philippines.
(XLS)

Table S4 Differentially expressed genes in *Xanthomonas oryzae* pv. *oryzae* strains when strain GD1358 (C5) from China is compared with strain PXO86 (P2) from the Philippines.
(XLS)

Table S5 Differential gene expression profiles of the genes related to the two-component systems (TCS) in three *Xanthomonas oryzae* pv. *oryzae* strains, PXO99 (P6) and PXO86 (P2) from the Philippines and GD1358 (C5) from China.
(XLS)

Table S6 Differential gene expression profiles of the genes related to chemotaxis and bacterial motility in three *Xanthomonas oryzae* pv. *oryzae* strains, PXO99 (P6) and PXO86 (P2) from the Philippines and GD1358 (C5) from China.
(XLS)

Table S7 Differential gene expression profiles of gums genes and gene involved in synthesis of exopolysaccharides and xylanase in three *Xanthomonas oryzae* pv. *oryzae* strains, PXO99 (P6) and PXO86 (P2) from the Philippines and GD1358 (C5) from China.
(XLS)

Table S8 Differential gene expression profiles of *hrp* genes in three *Xanthomonas oryzae* pv. *oryzae* strains, PXO99 (P6) and PXO86 (P2) from the Philippines and GD1358 (C5) from China.
(XLS)
