# Supplementary figures and images for: Comparative Transcriptome Profiling Reveals Different Expression Patterns in Xanthomonas oryzae pv. oryzae Strains with Putative Virulence-Relevant Genes
Source: PLoS One. 2013 May 29;8(5):e64267. doi: 10.1371/journal.pone.0064267 (PMC3667120; doi:10.1371/journal.pone.0064267)

## Slide 1
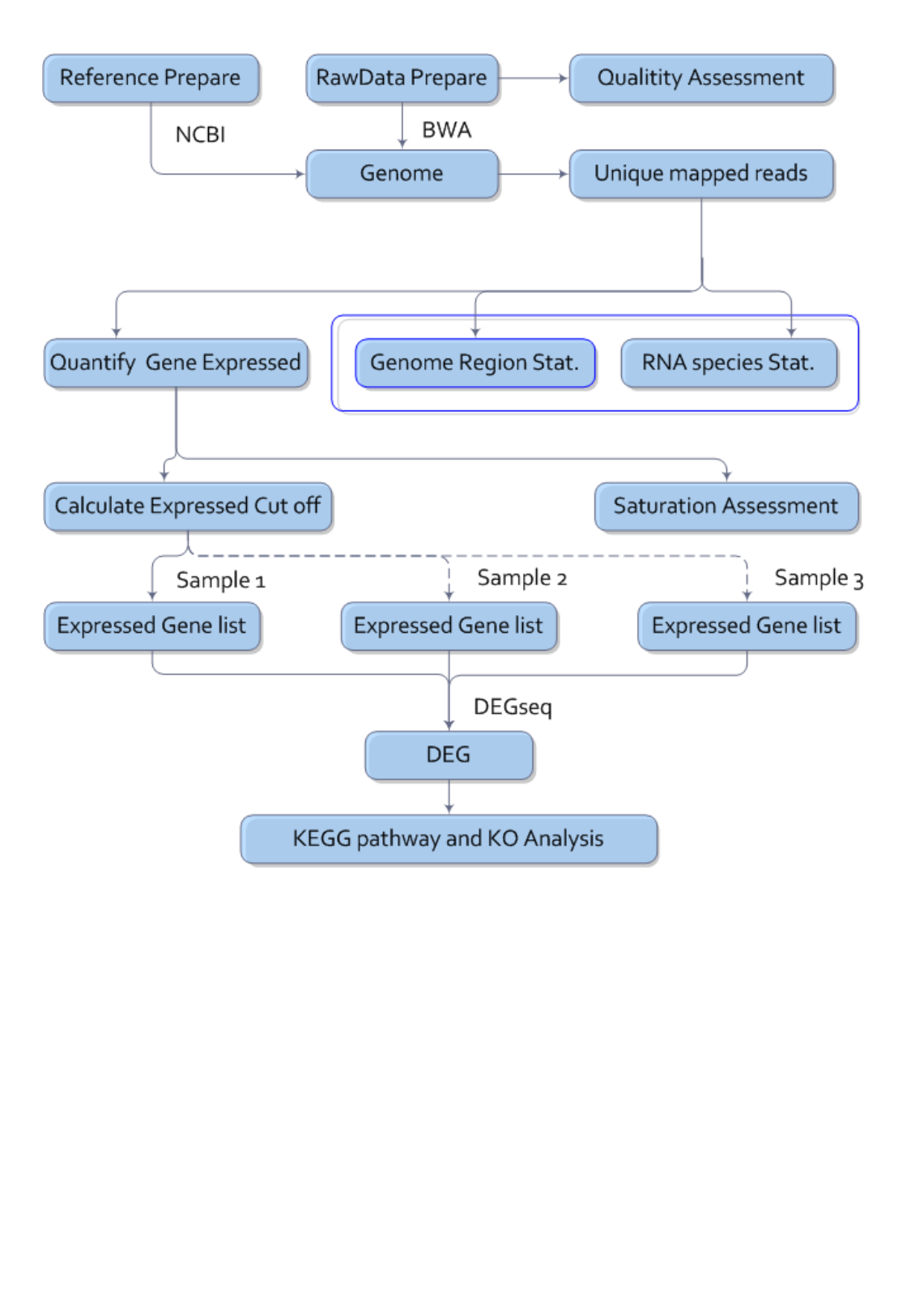

Supplement: Figure S1 — The analysis workflow for RNA-seq data based on mapping reads to the reference genome sequence, quantifying the gene expressed, identifying DEGs and categorizing DEGs by Gene Ontology. (PPT) [file pone.0064267.s001.ppt]

## Slide 1
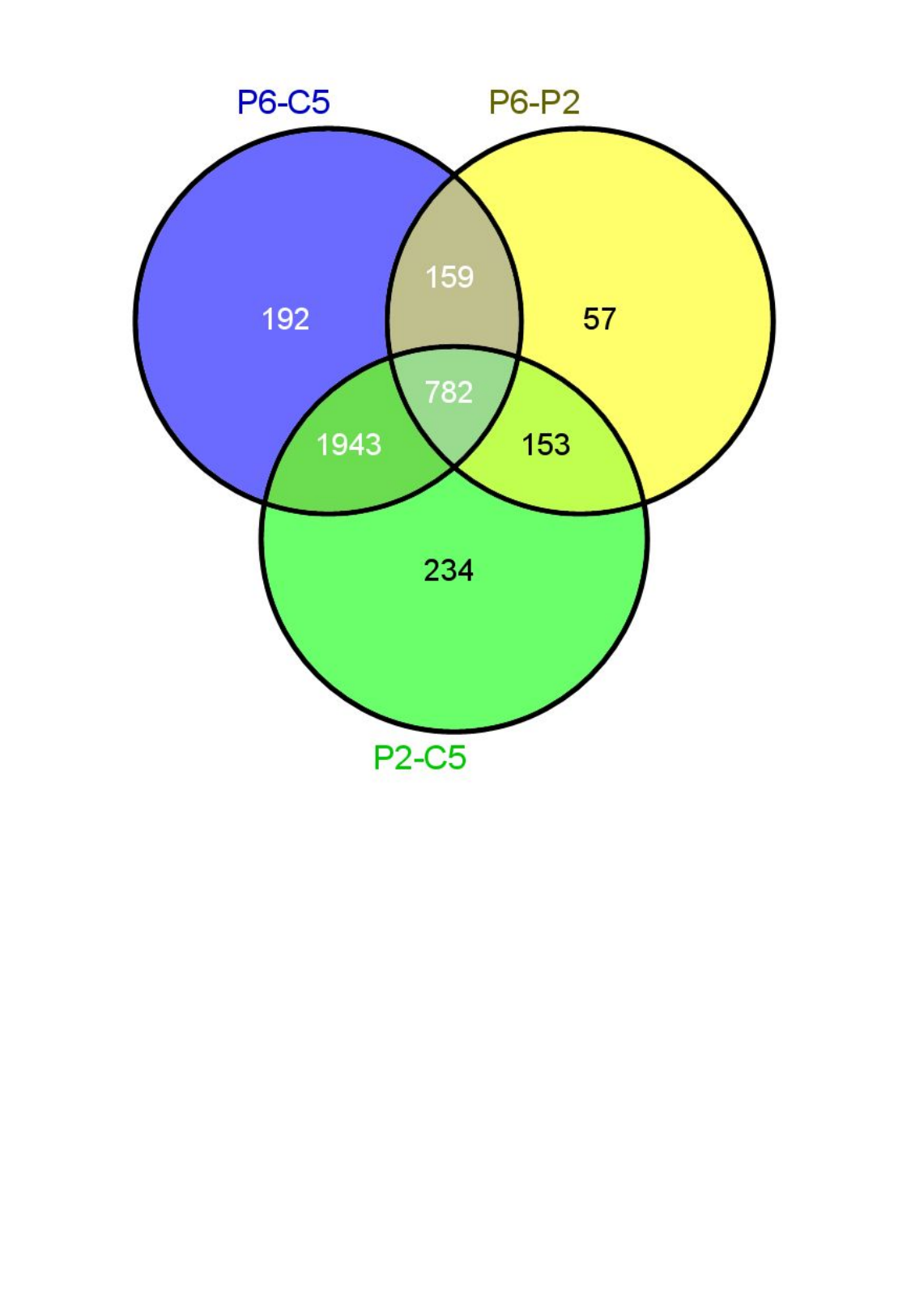

Supplement: Figure S2 — The Venn diagram of all the DEGs between each of the comparisons (P6 vs. C5, P6 vs. P2, P2 vs. C5). P6, P2 and C5 indicate Xanthomonas oryzae pv. oryzae strain PXO99, PXO86, and GD1358, respectively. (PPT) [file pone.0064267.s002.ppt]
