# Supplementary material for: Comparative Transcriptome Profiling Reveals Different Expression Patterns in Xanthomonas oryzae pv. oryzae Strains with Putative Virulence-Relevant Genes
Source: PLoS One. 2013 May 29;8(5):e64267. doi: 10.1371/journal.pone.0064267 (PMC3667120; doi:10.1371/journal.pone.0064267)
Supplement: Figure S3 — The expression patterns of 33 genes in three Xanthomonas oryzae pv. oryzae strains, PXO99 (P6) and PXO86 (P2) from the Philippines and GD1358 (C5) from China. (PPT) [file pone.0064267.s003.ppt]

## Slide 1
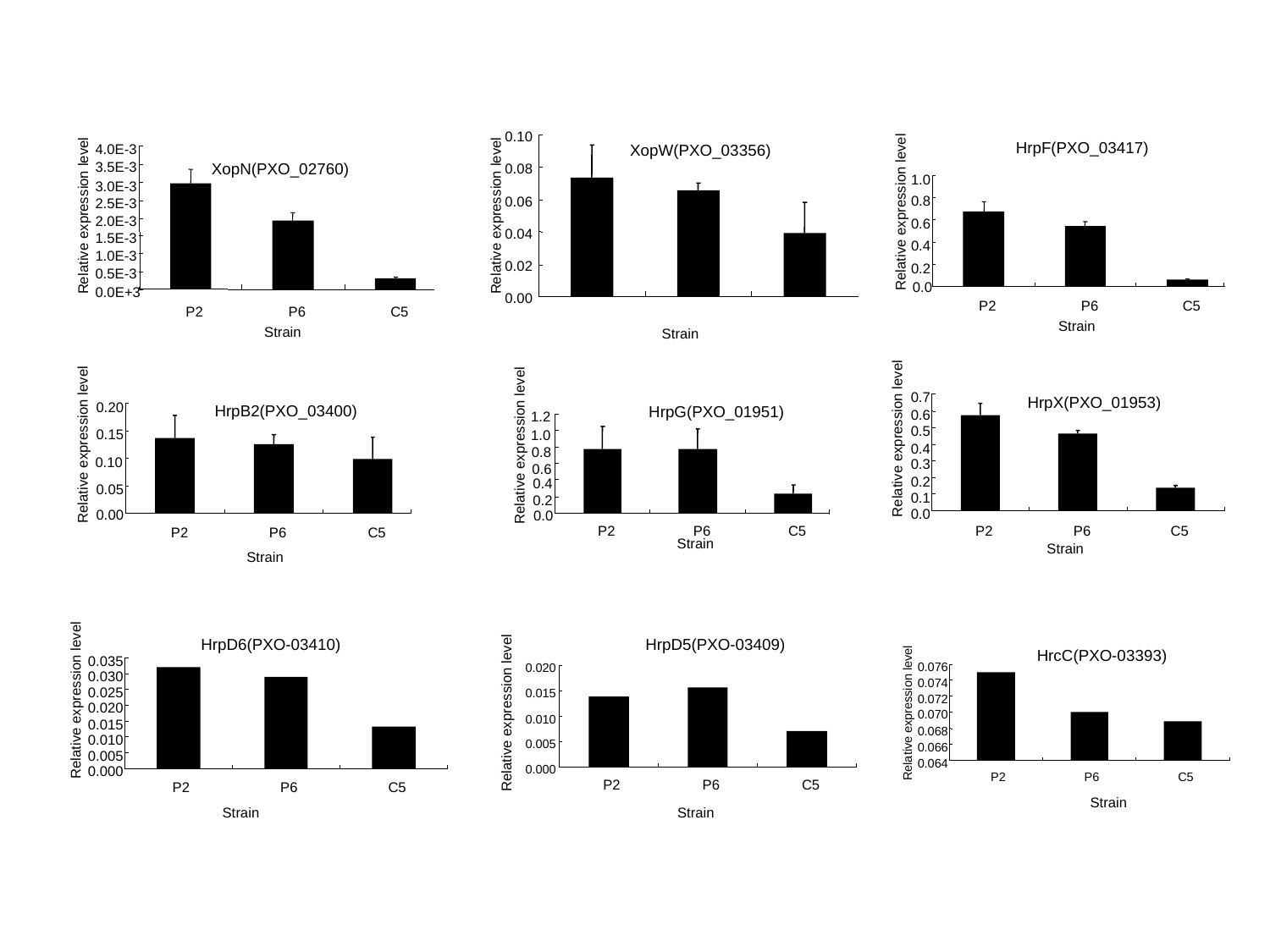

0.10
4.0E-3
3.5E-3
XopN(PXO_02760)
3.0E-3
2.5E-3
Relative expression level
2.0E-3
1.5E-3
1.0E-3
0.5E-3
0.0E+3
P2
P6
C5
Strain
HrpF(PXO_03417)
XopW(PXO_03356)
0.08
1.0
0.8
0.06
Relative expression level
Relative expression level
0.6
0.04
0.4
0.02
0.2
0.0
0.00
P2
P6
C5
Strain
Strain
HrpX(PXO_01953)
0.7
0.20
HrpB2(PXO_03400)
HrpG(PXO_01951)
0.6
1.2
0.5
0.15
1.0
Relative expression level
Relative expression level
Relative expression level
0.4
0.8
0.10
0.3
0.6
0.2
0.4
0.05
0.1
0.2
0.0
0.00
0.0
P2
P6
C5
P2
P6
C5
P2
P6
C5
Strain
Strain
Strain
HrpD6(PXO-03410)
HrpD5(PXO-03409)
HrcC(PXO-03393)
0.076
0.074
0.072
Relative expression level
0.070
0.068
0.066
0.064
P2
P6
C5
0.035
0.020
0.030
0.025
0.015
Relative expression level
0.020
Relative expression level
0.010
0.015
0.010
0.005
0.005
0.000
0.000
P2
P6
C5
P2
P6
C5
Strain
Strain
Strain

## Slide 2
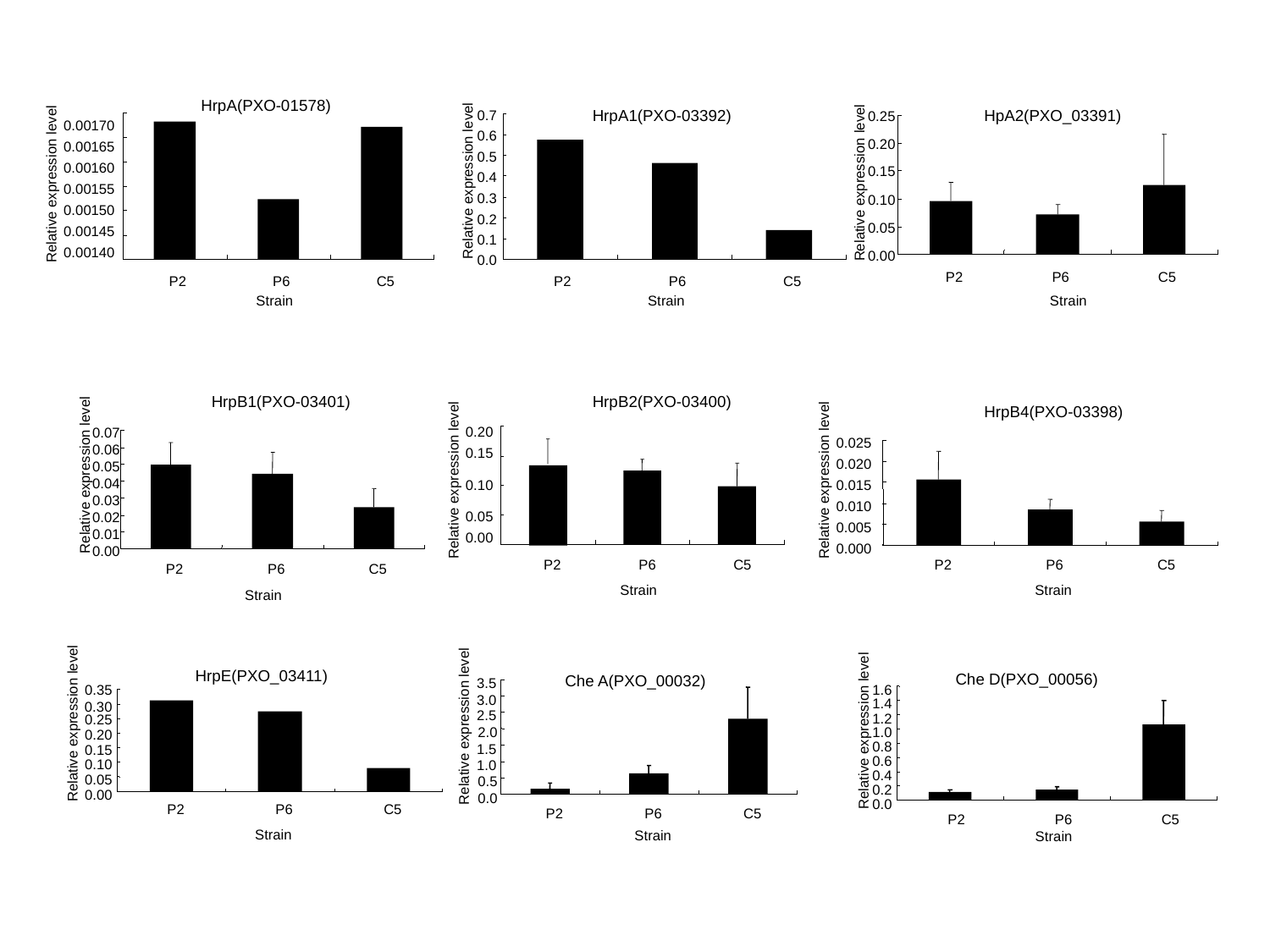

HrpA(PXO-01578)
HrpA1(PXO-03392)
HpA2(PXO_03391)
0.7
0.25
0.00170
0.6
0.20
0.00165
0.5
0.00160
0.15
0.4
Relative expression level
Relative expression level
Relative expression level
0.00155
0.3
0.10
0.00150
0.2
0.05
0.00145
0.1
0.00140
0.00
0.0
P2
P6
C5
P2
P6
C5
P2
P6
C5
Strain
Strain
Strain
HrpB1(PXO-03401)
HrpB2(PXO-03400)
HrpB4(PXO-03398)
0.20
0.07
0.025
0.06
0.15
0.020
0.05
Relative expression level
Relative expression level
Relative expression level
0.04
0.10
0.015
0.03
0.010
0.05
0.02
0.005
0.01
0.00
0.000
0.00
P2
P6
C5
P2
P6
C5
P2
P6
C5
Strain
Strain
Strain
Relative expression level
HrpE(PXO_03411)
Che D(PXO_00056)
Che A(PXO_00032)
3.5
0.35
1.6
3.0
1.4
0.30
2.5
1.2
0.25
Relative expression level
Relative expression level
2.0
1.0
0.20
0.8
1.5
0.15
0.6
0.10
1.0
0.4
0.05
0.5
0.2
0.00
0.0
0.0
P2
P6
C5
P2
P6
C5
P2
P6
C5
Strain
Strain
Strain

## Slide 3
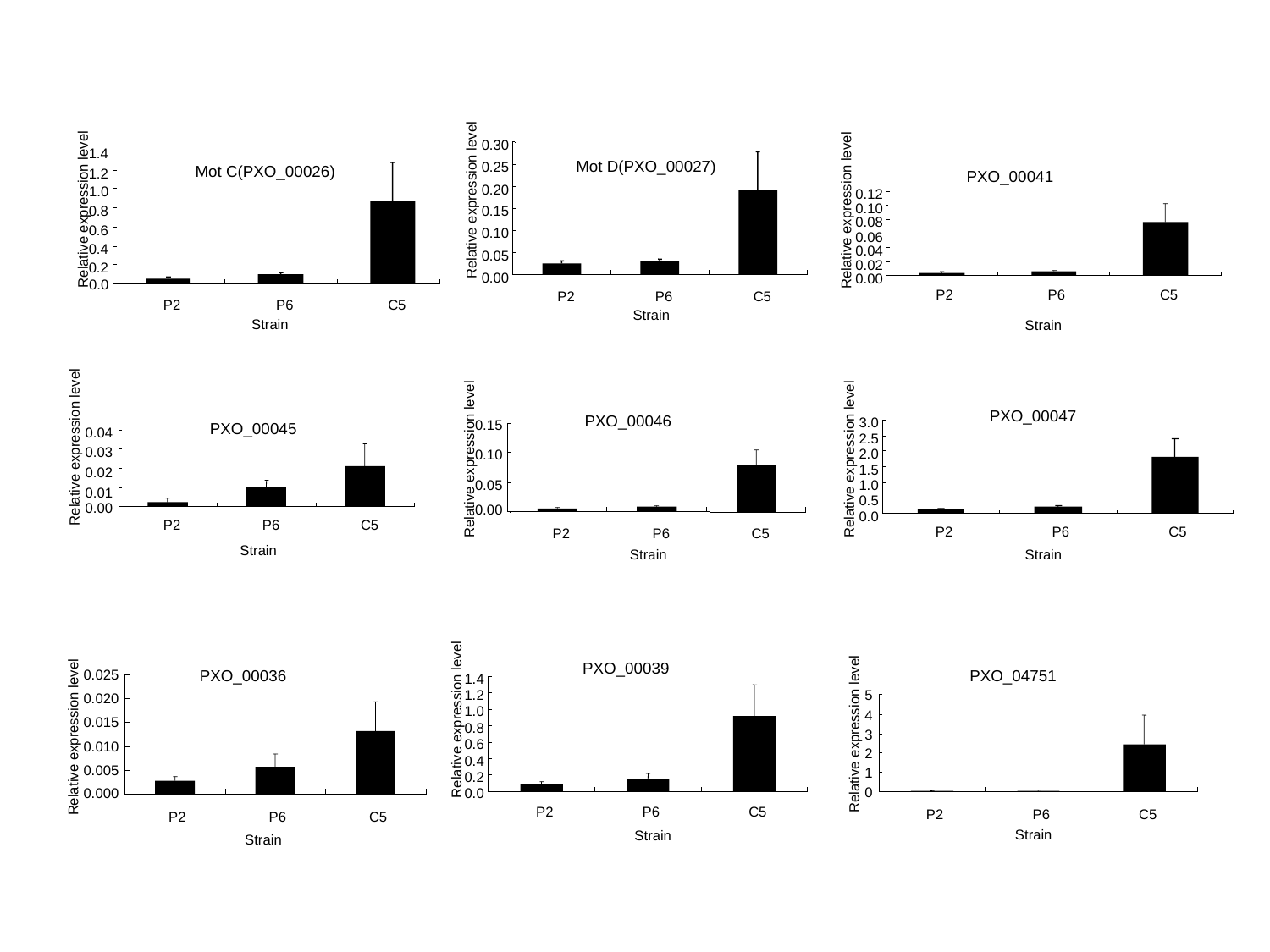

0.30
1.4
Mot D(PXO_00027)
0.25
Mot C(PXO_00026)
1.2
0.20
1.0
Relative expression level
Relative expression level
0.8
0.15
0.6
0.10
0.4
0.05
0.2
0.00
0.0
P2
P6
C5
P2
P6
C5
Strain
Strain
PXO_00041
0.12
0.10
Relative expression level
0.08
0.06
0.04
0.02
0.00
P2
P6
C5
Strain
PXO_00047
PXO_00046
3.0
0.15
PXO_00045
0.04
2.5
Relative expression level
0.03
2.0
0.10
Relative expression level
Relative expression level
1.5
0.02
0.05
1.0
0.01
0.5
0.00
0.00
0.0
P2
P6
C5
P2
P6
C5
P2
P6
C5
Strain
Strain
Strain
PXO_00039
0.025
PXO_00036
PXO_04751
1.4
1.2
5
0.020
1.0
4
Relative expression level
0.015
0.8
Relative expression level
3
Relative expression level
0.6
0.010
2
0.4
0.005
1
0.2
0
0.000
0.0
P2
P6
C5
P2
P6
C5
P2
P6
C5
Strain
Strain
Strain

## Slide 4
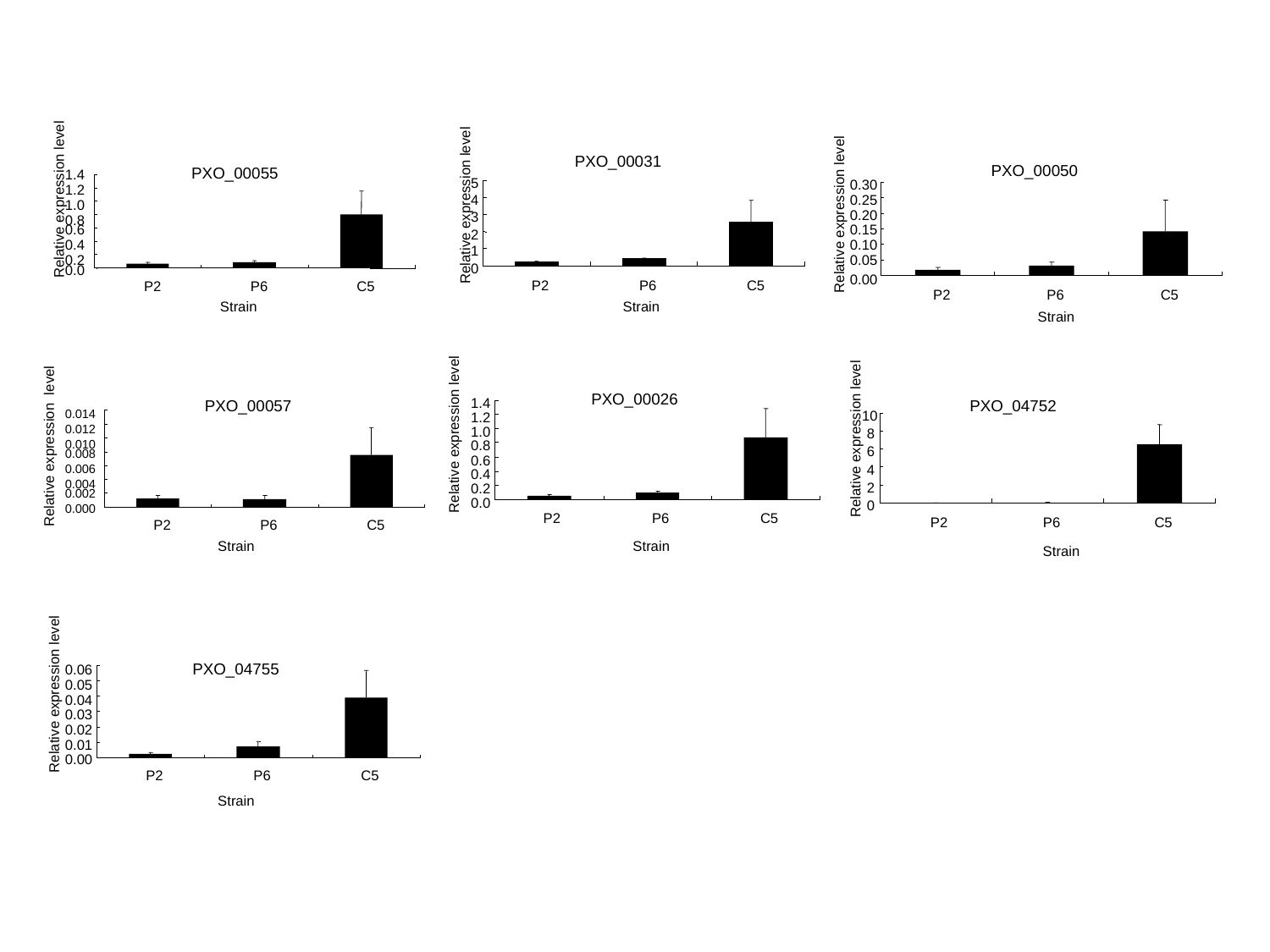

PXO_00031
PXO_00050
PXO_00055
1.4
5
0.30
1.2
Relative expression level
0.25
4
1.0
Relative expression level
Relative expression level
0.20
3
0.8
0.6
0.15
2
0.4
0.10
1
0.2
0.05
0
0.0
0.00
P2
P6
C5
P2
P6
C5
P2
P6
C5
Strain
Strain
Strain
PXO_00026
1.4
PXO_00057
PXO_04752
0.014
10
1.2
0.012
1.0
8
Relative expression level
Relative expression level
0.010
0.8
Relative expression level
6
0.008
0.6
4
0.006
0.4
0.004
2
0.2
0.002
0.0
0
0.000
P2
P6
C5
P2
P6
C5
P2
P6
C5
Strain
Strain
Strain
PXO_04755
0.06
0.05
Relative expression level
0.04
0.03
0.02
0.01
0.00
P2
P6
C5
Strain
